# Textile Pattern Design in Thermal Vision—A Study on Human Body Camouflage

**DOI:** 10.3390/ma14164364

**Published:** 2021-08-04

**Authors:** Catarina Pimenta, Carla Costa Pereira, Raul Fangueiro

**Affiliations:** 1 Centre for Textile Science and Technology, University of Minho, Campus de Azurém, 4800-058 Guimarães, Portugal; 2 Faculty of Architecture, University of Lisbon, Rua Sá Nogueira, 1349-063 Lisbon, Portugal; carlota.morais@gmail.com; 3 Department of Mechanical Engineering, University of Minho, Campus de Azurém, 4800-058 Guimarães, Portugal; rfangueiro@dem.uminho.pt

**Keywords:** fashion design, textile material, textile pattern, thermal camouflage, biomimetic

## Abstract

This paper reports on a new approach to the creation process in fashion design as a result of the exploitation of thermal camouflage in the conceptualization of clothing. The thermal images’ main variation factors were obtained through the analysis of their color behavior in a (diurnal and nocturnal) outdoor beach environment, with the presence and absence of a dressed human body (through the use of a thermal imaging camera), such as the analysis of textile materials in a laboratory (simulating the captured outdoor atmospheric temperatures and those of the model’s skin using the climatic chamber and the thermal manikin). The combination of different patternmaking, sewing and printing techniques in textile materials, along with the study of the camouflage environment and the human body’s variation factors, as well as the introduction of biomimetic-inspired elements (chameleon’s skin), enabled the creation of a clothing design process with innovative de-sign elements which allow us to thermally camouflage the human body and take clothing beyond the visible spectrum in a functional and artistic way.

## 1. Introduction

In the creation of clothing, several elements must be considered in the design process, in order to achieve a greater understanding of the concept that is intended to develop (e.g.,: the silhouette; the function; the proportion and line; the details; the printing and ornamentation; the color; the material; etc.) [1]. In this context of creation, it is possible to observe the purpose of unconventional materials [2], such as intelligent materials or materials that are changeable to stimuli (e.g. light, heat, etc.), adaptable to the environment in an interactive and intelligent way [3]. Collaborations between researchers in the areas of nanotechnology, biotechnology and digital technology allow the reaching of this textile innovation [4]. Clothing is also related to the communication component, the influence on individuals, without the use of words [5], allowing the transmission of innumerable meanings, as to suggest, to insinuate, to lie, among others [6].

The concealment and disclosure of the body can be associated to cultural and religious issues, such as the example of the use of the burqa [7]. However, in contemporary western society, aspects related to personal needs and desires reveal another reality, such as the problematic of technological surveillance of human activity, where visible and invisible worlds are observed, sensed and tested through technology. The exploration of a hypervisibility in clothing that allows to react against common sense visibility [8], it has been an emerging trend of “illusion wear” (optical illusions in clothing), and which allow not only the manipulation of the proportion/shape of the human body [9] such as changing one’s identity. Some examples of projects related to optical camouflage, thermal camouflage and pattern manipulation in the textile material are observed in the development of clothing, namely: the “Invisibility cloak” by Susumu Tachi; the “Digital skins” by designer Nancy Tibury [10]; the conceptual project of clothing with different patterns visible in thermal image [11].

### 1.1. Biomimetic

Human beings are surrounded by geniuses on planet Earth [12], where to Nature is associated the role of final designer while coworker, providing diversified sources of inspiration, for the creation of new materials in order to obtain greater performance, to respond to the needs and capacity of modern industry [13]. Examples of commercial applications are the development of innovative nanomaterials and nanodevices based on bacteria, plants and animals [14]. Biomimetic inspiration is combined with the potential for innovation, the integration of technology with natural ecosystems, as well as the possibility of redesigning the way how humanity relates with and inhabits the planet [15]. Thus, the exploration of the biological structure of living beings allows new relationships between materiality, form and function, correlated with multidisciplinary approaches [16], being possible the exploration of the fusion of different concepts observable in Nature with the aim of creating multifunctional materials [17]. Therefore, the study of the functionality coming from the surface and structure of the animals is revealed as a field of observation and learning in great growth currently. Examples of surface studies are materials with optical properties, superhydrophobic, anti-wear, anti-fog and drag reduction, among others [18]. Consequently, the study of the structural component appears as a fundamental element in the addition of innovative properties to materials in a context where the fashion designer resembles an engineer, in an attempt to create portable environments in clothing [19]. Innovation with inspiration in Nature emerges as a support for the creation of new textile products adapted to various purposes, as illustrated by the example of Velcro^®^, inspired by structural principles from the burdock plant burrs [20]. In the animal world, we can observe the exploration of the adhesion mechanism in the foot of the gecko (in its ability to scale different surfaces vertically), in the development of the vast field of nanotechnology and modern engineering, being some examples the following scientific studies/articles [21,22,23,24]. Beyond reptile skin, the shells of molluscs are also examples of natural structures noticeable in Nature, associating animals such as octopus, squid, cuttlefish as examples of inspiration for functional products based on mimetic properties related to camouflage and color change [25]. Thus, in an aspect of adaptation to wearable interactive devices, the “electronic skin” (e-skin) project is observed, which allows interactive color changes, derived from the study of the capacity of alteration and color change visualized in the chameleon’s skin [26]. Otherwise, the study of the structural colors present in some animals, derived from mechanisms with complex interactions between light and microstructures, have great potential for application in different industrial areas, such as textile [27], being an example the design of a transparent structural colored film, with inspiration in the optical transparency evident in the wings of the insect *Cephonodes hylas* that allows it to hide from enemies and camouflage in the environment [28], such as examples that link the nanotechnology and study/inspiration in structural color and optical properties present in the wings of the morpho butterfly [29,30,31] and the wings of the Rajah Brooke’s birdwing butterfly [32]. In this situation, the structure is a relevant principle for application in engineering, which may enable the creation of new more benign ways of obtaining color [33]. In the modern textile industry and engineering, as explained previously, examples of projects and materials based on the structure are observed, namely: the clothing designed by Donna Sgro with the textile material “Morphotex” inspired by the morpho butterflies; the textile material “FastSkin” from the Speedo brand, inspired by shark’s skin; the textile material “Geckskin”, inspired by the gecko’s feet; the “lotus effect” on textile surfaces, inspired by the lotus leaf. Yet, in the active camouflage aspect, the creation of dynamic soft textile surfaces is observed in a project led by Xuanhe Zhao, inspired by the behavior of the octopus’s skin [16]. Otherwise, the exploration of biomimetics and its relationship with nature is also observed in contexts of fashion installation, sending the viewer to a new reality, such as the example of the “Biopiricy” (Autumn/Winter, 2014) and “Aeriform” (Autumn/Winter, 2017) collections, by designer Iris Van Herpen, also relating that the “negative space” of fashion, which surrounds clothing, also raises issues beyond the materiality of clothing. The film is considered as the future, in terms of a tool for fashion exhibition [34] and the fusion between technology and fashion will allow an improvement in terms of interactive and expressive possibilities while creating a poetic use/experience [35]. In addition, the effects of textile surface, of materials that change color or shape still have a character of exhibition that involves a theatrical and intimate dialogue, without the need for specific performance techniques [36]. 

### 1.2. Proposal

The main objective of this work refers to the development of a new clothing creation process with visible patterns in thermal images, in order to allow the thermal camouflage of the human body in an outdoor environment (diurnal and nocturnal), previously idealized. New design elements will be introduced in the clothing creation process, through the exploration of the behavior of colors in thermal image of the environment, human body and textile material, as well as the combination of manual and digital drawing, and the exploration of biomimetics, with the introduction of the study/inspiration of the chameleon’s skin (animal associated with its facility of camouflage through the change of skin color, where the capacity appears initially in the literature through dermal chromatophores, and posteriorly by the adjustment active of a network of guanine nanocrystals that constitute the dermal iridophores [37,38]).

To sum up, it will be possible to observe that thermal vision and thermography can enable the development of several clothing design projects, such as the exploration of new paths in the creation process, in a functional aspect (e.g.,: military camouflage) or in an artistic and conceptual aspect (e.g.,: exhibition and performance contexts).

In this sense, the work is planned through the exploration of the thermal image, visible in different environments (outdoor and laboratory) through the use of a Testo 855 thermal imaging camera (Testo, Barcelona, Spain) in a spectrum range between 7.5–14 μm, with measurement option from −30 °C to 100 °C, emissivity value set to 0.95 (approximate emissivity value of the skin) and “Iron” color range selected. 

For the design of the clothing in an outdoor environment, a rocky place by the sea was selected in order to reduce possible changes in the landscape over time (Carreço beach, Viana do Castelo, Portugal).

For the laboratory environment, a FITOCLIMA 24000 EDTU walk-in climatic chamber was selected. The value of 65% humidity was applied, and the atmospheric temperature values captured outdoors with a HI9564 thermal hygrometer (HANNA) were reproduced, in which for the nocturnal simulation 18 °C of atmospheric temperature and for the diurnal simulation 24 °C atmospheric temperature was applied. To replace the human figure in the outdoor environment, a Newton 34-zone sweating thermal mannekin) was used, which was programmed with a temperature of 34 °C, from the ISO 15831 standard (procedure used to measure the thermal insulation of clothing on a thermal mannekin).

Thus, with the comparison and study of the results obtained from various thermal images of the textile materials, the human body and the environment (indoor and outdoor), it was possible to develop the new process of creation of the thermal camouflage clothing, through the selection of specific materials (conventional knitted 100% polyester fabric and copper metallic pigment) and techniques (printing, patternmaking of small structures and sewing) that allow the development of prototypes of thermal camouflage and later of the model’s thermal camouflage in the selected location, in a nocturnal environment and diurnal environment, in a moving and static way.

## 2. Materials and Methods

In a previous study carried out [39,40], thermal images were analyzed (in the “Iron” range of the thermal imaging camera), which allowed to elaborate different thermally visible color palettes were created, to assist the entire conceptual process, namely: the diurnal and nocturnal environment (with regard to the environment/body/clothing); and the laboratory environment (with regard to the environment/thermal manikin/textile material). The conceptual process was based on the perception of these resulting thermal colors in different selected environments (diurnal and nocturnal) in thermal image, in order to design clothing with finishing or structural surface treatments in the textile materials used according to these results to subsequently camouflage the best possible. Thus, the following materials with the following properties were selected for this study: (i) knitted fabric: jersey 100% polyester; white color; 153.39 g/m^2^; 0.42 mm; 26.9 Tex; 15 wales/cm; 20 courses/cm; 0.42 mm; 0.76 ε (without printing); 0.50 ε (with printed copper pigment); (ii) printed copper pigment (formula: 80 g Hydra Clear for Metallic (clear base of the brand Virus^®^, Water Based Inks, Bergamo, Italy) with 20 g Copper Powder (metallic powder of the brand Virus^®^, Water Based Inks, Bergamo, Italy).

Therefore, considering that in the diurnal environment there is a greater predominance of yellow, orange and magenta colors, in contrast to the nocturnal environment where there is a greater predominance of orange, magenta and blue colors, the study of patterns continued with the study and analysis of the colors of different types of materials and different types of sewing techniques (which are present in the paper [40], Figure 11 and Table 3, namely: (i) 100% polyester knitted fabric to obtain the yellow color; (ii) 100% polyester printed knitted fabric with copper pigment to obtain the orange color; (iii) application of embossed structures with fully fusible interlining to obtain the magenta color; and (iv) application of embossed structures with partially fusible interlining to obtain effects in the magenta and orange colors). Following the study of the data observed in the laboratory (climatic chamber), as well as in the outdoor (in the place of future camouflage), it was possible to observe that the properties of the textile materials used/tested (e.g., textile structure, composition, yarn linear density, mass per square meter, thickness, emissivity, thermal resistance, thermal conductivity), by themselves, are not enough to present or to guarantee high performance of thermal camouflage functionality of clothing. However, it was observed the extreme relevance of manipulation, control and study of the AIR element present between the surface of the textile material and the skin surface of the model’s body and the thermal mannequin, because this interferes with the interaction and stability of the color behavior of the image in thermal vision and the consequent performance of the textile material in thermal camouflage contexts. 

Consequently, the functionality of thermal camouflage and the creation of illusory effects on clothing can only be acquired through the combination of the properties of the textile material and with an efficient and creative design process, which enhances the visual characteristics present in the textile material in thermal vision, in its interaction with the human body that uses it and the environment that surrounds it. 

Thus, the study of design becomes extremely important for the textile material/clothing and textile pattern developed to perform its functionality in an efficient way, in the different perspectives that interact with the idealized thermal camouflage environment.

Therefore, the use of image overlay techniques (with the presence and absence of the human body), as well as the combination of a manual and digital drawing process, was essential for the understanding of the camouflage environment, the silhouette of the model (human body in a static and in a moving position) and the conceptualization of textile patterns which will possibly be applied to the materials used in the clothing to be created later. 

The desired camouflage colors are observed in the thermal image with the presence of body temperature. However, it is only with the absence of the model’s body in the thermal image that it becomes possible, by comparative method, to analyze all textures and patterns that can provide absence and presence of camouflage. The body presence in the thermal image allows, on the other hand, to project and design in the ideal proportion of the clothing, in order to obtain a better functionality. 

### 2.1. Development of Patterns

**Figure 1:**
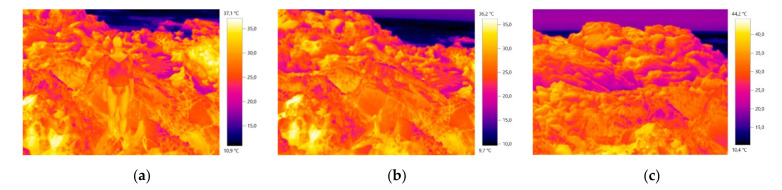
Thermal images of the base of the conceptual process of the diurnal prototype: (**a**) with the presence of the human body; (**b**) and (**c**) with the absence of the human body.

**Figure 2:**
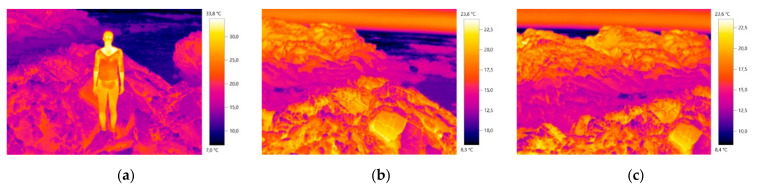
Thermal images of the base of the conceptual process of the nocturnal prototype: (**a**) with the presence of the human body; (**b**) and (**c**) with the absence of the human body.

**Figure 3:**
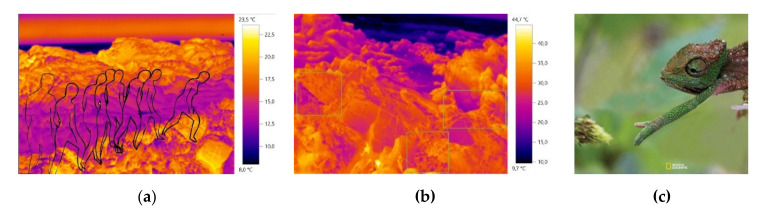
(**a**) Image of study of movement and silhouette; (**b**) Inspirational thermal image for the pattern modules M01 and M02; (**c**) Adapted chameleon-inspired photograph [41].

In the course of the investigation, it was verified that for the creation of design process, the image and the materials’ real color is not determinant in obtaining camouflage, being relevant another type of creation approach, more focused on the embossing of the material structure. In this sense, the study and inspiration of the chameleon’s skin behavior was also interconnected in the combination of the colors, patterns and structures of the design process for the camouflage outfit. The inspiration/study of the chameleon, focused on the observation of the behavior of the visible color on the skin (colors and dimensions of patterns) as well as the small embossments and texture observed (irregular shapes and different dimensions). Some images that supported the basis of the conceptual process can be observed below in Figure 1, Figure 2 and Figure 3.

**Figure 4:**
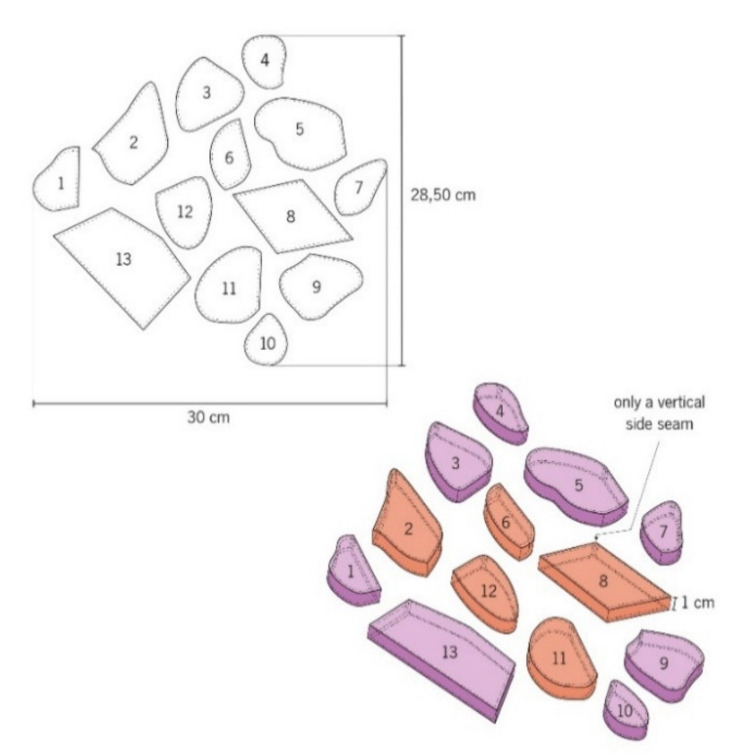
Technical drawing of the embossed structures of the pattern module M01.

**Figure 5:**
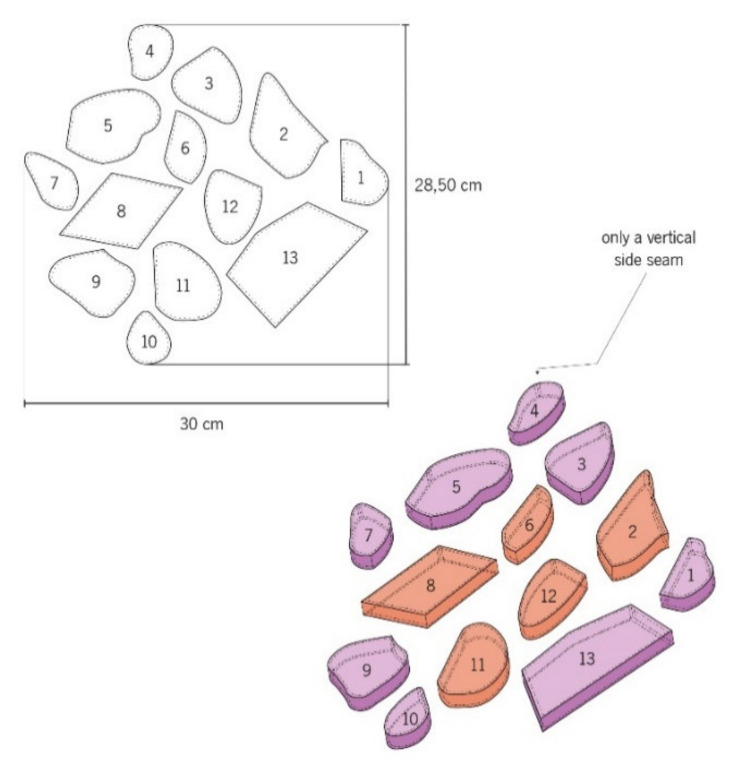
Technical drawing of the embossed structures of the pattern module M02.

Among the various experiments carried out, different types of modules were performed, namely:

- M01 and M02, with measures of 28.50 cm × 30 cm, for the study of the manipulation of the air between the textile material and the human body using the printing and sewing of embossed structures techniques (1 cm height) with total and partially fusible interlining (to obtain the magenta color and effects in the magenta/orange color in thermal image). The technical drawings of the modules M01 and M02 can be observed in Figure 4 and Figure 5. All small structures present an only vertical side seam, such as 0.5 cm of sewing value in all fittings. In addition, the fusible interlining was applied only on the side strip that raises and contours the shape (numbers: 2; 3; 8 and 9), as well as on the side strip and in the totality of the shape (numbers: 1; 4; 5; 6; 9; 10; 11; 12 and 13).

Subsequently, the air behavior of the M01 module was observed in laboratory, similarly to previous experiments [39,40].

**Figure 6:**
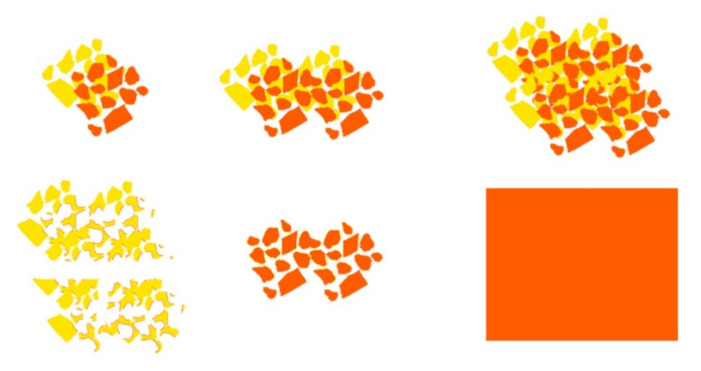
Assembly of different elements of the M03 module.

**Figure 7:**
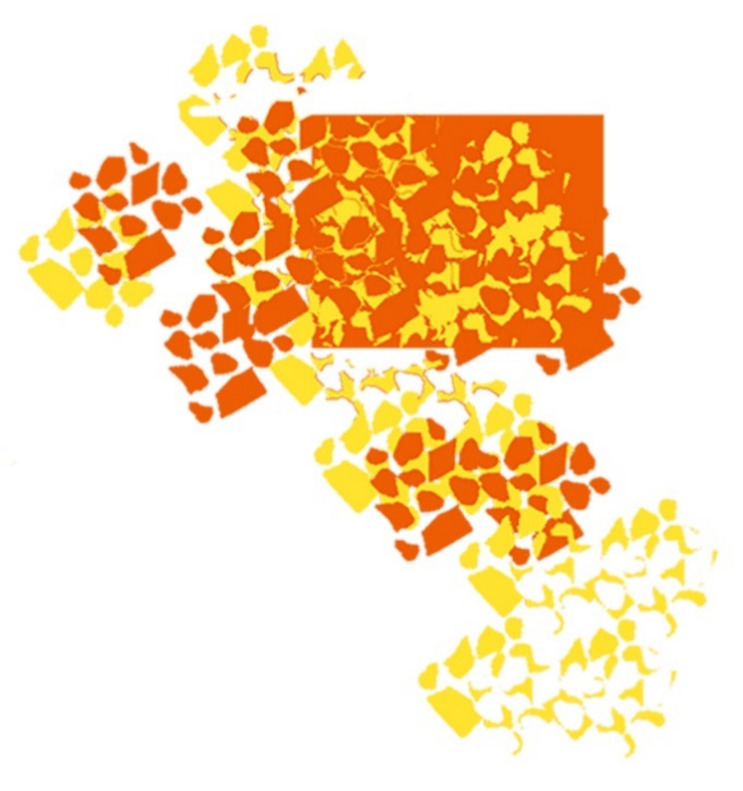
Final study of the M03 module.

- M03, M04 and M05—based on M01 and M02 modules, were created in order to be placed tight to the body through the combination of the printed copper pigment technique and absent printed pigment areas (to obtain the yellow and orange colors in thermal image). Techniques of overlapping, rotating and moving of the shapes present in M01 module and M02 module, Figure 6 and Figure 7, allowed to obtain a pattern with “pixelated” effect M03 (with measures of 50 cm × 50 cm), with abstract, asymmetric, irregular shapes and with different types of paint filling.

Sequentially, the M03 module was reflected, giving rise to the M04 module (with measures of 50 cm × 50 cm). The M05 module (with measurements of 60 cm × 35 cm) was created with the intention of facilitating the subsequent process of sewing and patternmaking of the mask, keeping the original fitting of the M03 module on the front and creating another design for fitting with the M04 module, on the back.

- The M06 module was also created (with measurements of 50 cm × 50 cm), to be placed tight to the model’s body using the printed copper pigment technique (to obtain the orange color in thermal image).

Once the modules were conceptualized, it was possible to develop two thermal camouflage prototypes (full-length overalls, in order to cover the body in its totality and be adaptable to the body movement): one for a diurnal environment and another for a nocturnal environment.

### 2.2. Development of Clothing Prototypes

In the diurnal prototype, the following pattern modules were applied: i) two pattern modules with small sewn embossed structures, with copper pigment, to provide orange and magenta colors in thermal image, namely: the “original” M01 placed on the front and the “reflected” M02 on the back of the prototype; (ii) three pattern modules with two colors visible in thermal image (yellow and orange), created through the absence and application of copper pigment on the knitted fabric, namely: the “original” M03 applied on the front of the prototype; the “reflected” M04 applied on the back; and the M05 of the mask that includes the front and the back; (iii) a pattern module with full color fill M06, with copper pigment to provide orange color in thermal image. Otherwise, in the nocturnal prototype, the following pattern modules were applied: (i) an “original” M01 pattern module with small sewn embossed structures, with copper pigment, to provide orange and magenta colors; (ii) and a pattern module with full color fill M06, with copper pigment to provide the orange color in thermal image. 

Furthermore, in the conceptualization process, different drawing materials and software such as V 3.7 (Testo), Adobe Photoshop and Adobe Ilustrator were used. After the final conceptualization process, the printing, patternmaking and sewing processes of all prototypes were performed, with specific materials for both practices.

## 3. Results

For the conceptualization of clothing with thermal camouflage properties, the textile material and techniques chosen must adapt to the color present in the environment that will surround the model’s body in the thermal image, as well as to the patterns and textures that surround it, and must present a similar color to the thermal result, if the aim is to camouflage. In contexts of thermal camouflage, the increase of the performance of the textile material and its thermal and emissive properties, is only acquired with the combination of the creative design process where the air component is extremely important in exploring the dynamics of the textile material with the body and the environment, since this element (AIR) allows the change and manipulation of colors on the body and silhouette of the model in thermal vision, emerging as a relevant and innovative element for design, in addition to temperature and thermal vision.

### 3.1. The Modules of the Conceptualized Patterns

#### 3.1.1. Combination of the Printing Technique with Patternmaking of Embossed Structures

**Figure 8:**
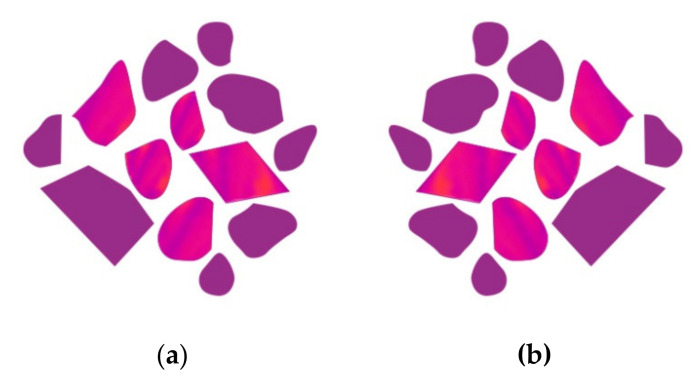
The pattern modules conceptualized with embossed structures and printed cooper pigment: (**a**) M01; (**b**) M02.

Figure 8 shows the final result of the illustration of the M01 (original) and M02 (reflected) module conceptualized, making it possible to observe the conjugation of the magenta color (derived from a fully fusible interlining structure) with effects of magenta and orange colors (derived from a partially fusible interlining structure). 

**Figure 9:**
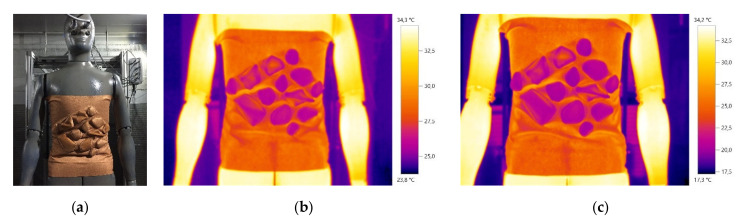
The M01 module tested in the laboratory: (**a**) Photography; (**b**) Thermal image of the diurnal simulation; (**c**) Thermal image of the nocturnal simulation.

The color variations in the thermal image of the M01 module observed in the laboratory, are shown in Figure 9, as well as the module’s sharpness and the ability to control and manipulate the air through small embossed structures. The desired colors for the Design conception of the camouflage prototype are observed in almost all structures, materials and techniques applied (100% polyester printed knitted fabric with copper pigment, with a combination of sewn embossed structures, fully and partially fusible interlining), in both diurnal and nocturnal simulations, previously tested. 

#### 3.1.2. Printing as Only Applied Technique

**Figure 10:**
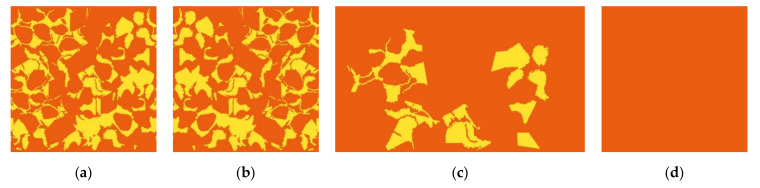
Pattern modules conceptualized with printed copper pigment: (**a**) M03; (**b**) M04; (**c**) M05; (**d**) M06.

Figure 10 shows the final result of the conceptualized modules M03, M04, M05 with the combination of yellow and orange colors derived from the absence and presence of copper pigment, as well as the M06 module, only with a single color printing, in copper pigment. 

#### 3.1.3. Application of the Modules in the Final Thermal Camouflage Prototypes

**Figure 11:**
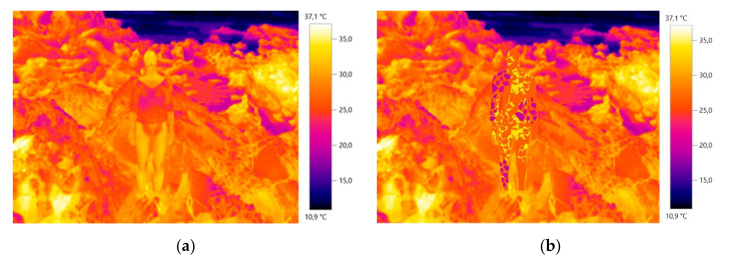
The diurnal thermal camouflage prototype conceptualized: (**a**) Base thermal image without the Design elaboration for the camouflage; (**b**) Base thermal image with the prototype illustration.

**Figure 12:**
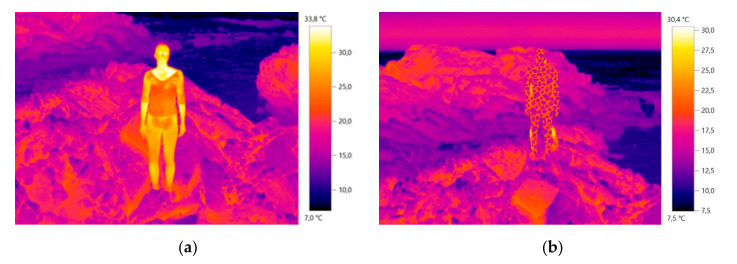
The nocturnal thermal camouflage prototype conceptualized: (**a**) Base thermal image without the Design elaboration for the camouflage; (**b**) Base thermal image with the prototype illustration.

Figure 11 and Figure 12 show a thermal image of the model in the future camouflage environment and illustrations of the thermal camouflage prototypes for each environment: diurnal (with modules M01, M02, M03, M04, M05 and M06) and nocturnal (with modules M01 and M06).

**Figure 13:**
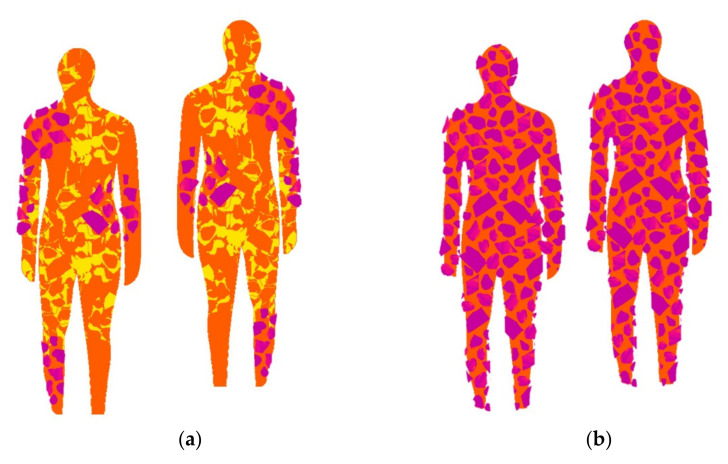
The conceptualized thermal camouflage prototypes: (**a**) Illustration of the front and back of the diurnal thermal camouflage prototype; (**b**) Illustration of the front and back of the nocturnal thermal camouflage prototype.

Figure 13 shows the final illustrations of the front and back applied to the conceptualized thermal camouflage prototypes (diurnal and nocturnal) and respective pattern modules, based on the dimensions and silhouette of the model’s body.

**Figure 14:**
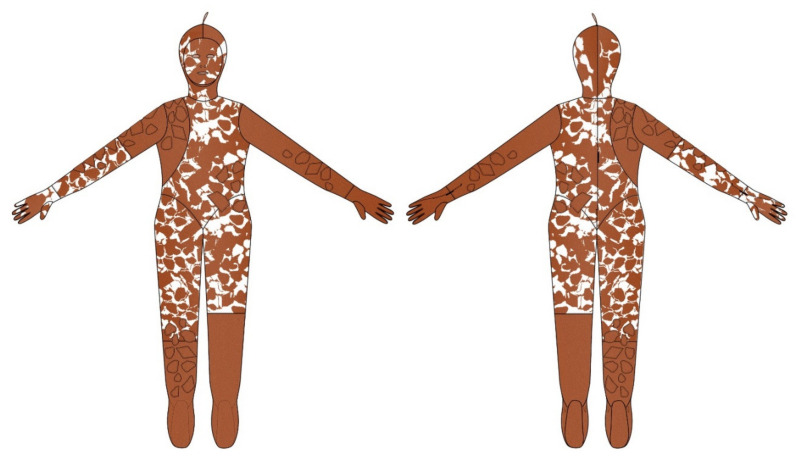
Technical drawing illustrated of the diurnal thermal camouflage prototype.

**Figure 15:**
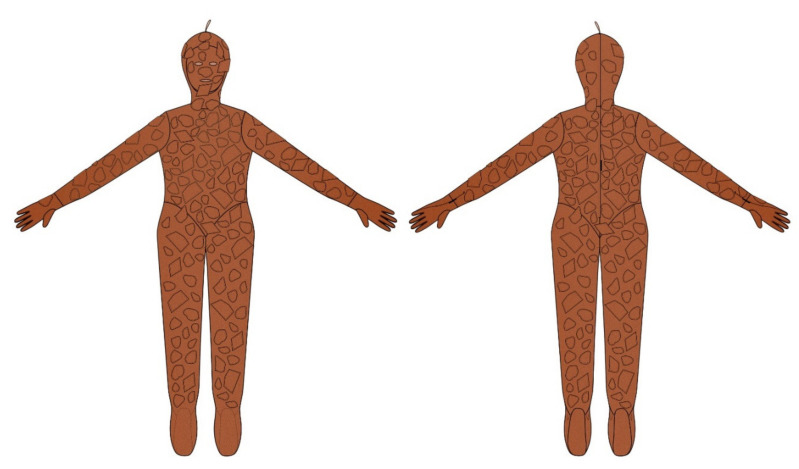
Technical drawing illustrated of the nocturnal thermal camouflage prototype.

The technical drawings of the front and back of both conceptualized prototypes can be observed in Figure 14, relative to the diurnal thermal camouflage prototype and in Figure 15, relative to the nocturnal thermal camouflage prototype. Both prototypes feature a full-length overall tight to the body, with sleeves and long trousers, with a mask, gloves and socks incorporated. In the eyes, nose and mouth area, openings were applied. In the center of the back, a zipper was applied as well as an invisible zipper on both gloves and in the finishing of the sleeves. The cuts, printing and patternmaking techniques were combined in different way, in the prototypes to obtain diurnal and nocturnal thermal camouflage, as previously analyzed, in this article. 

**Figure 16:**
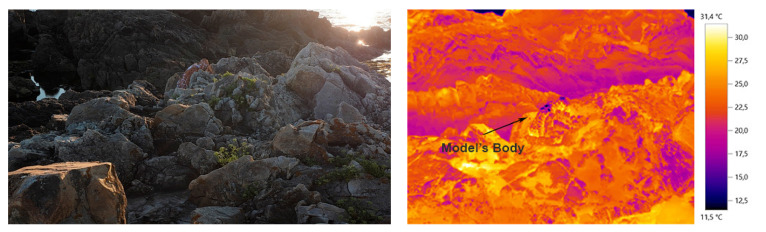
The conceptualized thermal camouflage prototype for the idealized diurnal environment [42].

**Figure 17:**
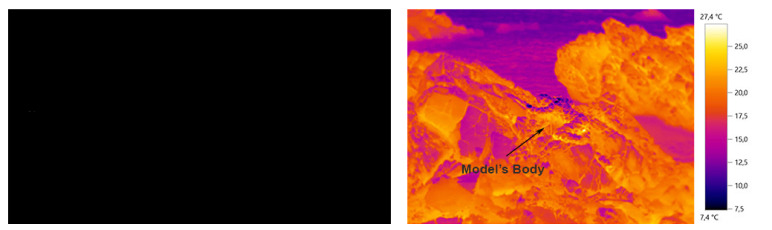
The conceptualized thermal camouflage prototype for the idealized nocturnal environment [42].

To sum up, the results of the final thermal tests carried out in the idealized thermal camouflage environment, with the diurnal and nocturnal thermal camouflage prototypes can be observed bellow in Figure 16 and Figure 17, taken from [42].

## 4. Discussion

The results obtained in the process of conceptualization of the prototypes and pattern modules allowed us to verify that it is possible to obtain and apply the thermal camouflage functionality in clothing, through the interconnection of the study of the different variants that involve the human body (female model), the environment (Carreço beach) and clothing (textile materials with different emissivity, combined with printing and patternmaking techniques). The increase in the performance and functionality of thermal camouflage of the textile material is obtained through the conjugation/application of the study and conceptual process of Design, exploring the clothing and the textile pattern in a global way, interconnected with the human body and the environment, where the air appears as a fundamental element in the control and stability of color in thermal image. Thus, the properties of textile materials (e.g., textile structure, composition, yarn linear density, mass per square meter, thickness, emissivity, thermal resistance, thermal conductivity), are not sufficient to enhance the functionality of thermal camouflage, since the material has to adapt to the surrounding visual environment, to its atmospheric conditions, as well as to the silhouette and temperature of the human body.

Therefore, in the conceptual design process for clothing with camouflage functionality, the following are relevant:

- The creation of a dynamic of colors in thermal image by the textile materials;
- The introduction of elements inspired by biomimetics in the design conception (e.g., chameleon skin);
- The study of the thermal image variation factors (atmosphere; human body; clothing/textile material; thermal imaging camera) and associated color palettes. In this case, the selection of another color palette (in addition to the “iron” range used in this work) will not alter the final result of thermal camouflage in clothing; however, it could provide less contrast and perceivability between the colors and temperatures captured by the thermal imaging camera in the selected color palette, which may make it difficult for the designer to work, when designing the clothing and, consequently, studying the color behavior visualized in the thermal image (either relative to the body, environment or textile material and techniques applied to it). Thus, in this parameter, it is up to the designer to reflect and choose the best method and color palette to apply.

In general terms, it was found that the thermal image with the presence of the model’s body (with and without movement) should be used as a base model and as a start for the design conception, for presenting the colors and textures intended for the camouflage environment and compare the study of the thermal image with absence of the model’s body, in order to verify all existing textures in the desired environment for the camouflage. In this way, it is essential to study the entire environment, all the representative details in the image (e.g., textures, materials, mechanisms…), reflecting and envisioning the behavior of the human body at the time of camouflage, in a military or performance context. The combination of biomimetics, the study of the diversity of mechanisms present in nature, can reinforce the effectiveness of functionality, as was observed in this study with the interconnection of the chameleon skin structure.

Otherwise, the use of new design elements in the clothing creation process (e.g.,: thermal vision, color of the thermal image, temperature, air and thermal and emissive characteristics of the materials) such as the combination of conventional materials (polyester knitted fabric), metallic pigments (copper) and sewing and patternmaking techniques (embossed structures), solves the reach of a possible diurnal and nocturnal thermal camouflage, namely: for functional purposes (for example, for military users, with a view to the total camouflage of the human body) or for artistic purposes (in exhibition and performance contexts, with the exploration of new design elements freely, with a view to the creation of emotions and new perspectives in the visualization of clothing). 

On the other hand, the impact of the varying textile patterns on the proposed clothing prototypes is very evident because the design and proportion of the pattern as well as the combination of different techniques (printing and sewn embossed structures) allow an interactive response between the temperature of the human body and the outdoor environment to which it is exposed, where the air and its absence, such as the combination of printing/pigment and its absence in the proposed pattern design enhances the response, the performance, the thermal camouflage functionality.

In this study, the 1 cm high embossed structures tested, in conventional knitted fabric printed with copper metallic pigment and with application of fusible interlining, potentiated another visible color in a thermal imaging camera (the magenta color), which without the introduction of this structure, the knitted fabric would present, adjusted to the human skin/body, the orange color (with printing) and the yellow color (without printing) in the thermal image. At this point, the structuring and manipulation of the air existing between the clothing and the human body/skin has a great impact on clothing, on the behavior of the textile material, on the conceptualized shapes and patterns and how these will later be seen in the thermal imaging camera. The introduction of different heights and dimensions in the textile structure, with the exploration of the elevation of the textile form, from the conceptual phase to the patternmaking and sewing phase, combining more or less irregular shapes, with greater or lesser dimensions, can and should be tested, in order to enable the study and control of more visible colors in thermal image, in search of creating new ways to achieve thermal camouflage. At this point, the introduction of the thermofixation technique, in a mechanical way, can help, facilitate and enable the development of a future structure.

Therefore, in addition to the application of surface design changes, the impact of varying textile patterns in the context of future work can be enhanced by using the introduction of new textile materials, which were not possible to be tested in this work, being an example the introduction and adaptation of smart materials and/or reactive materials, namely: thermochromic materials (which interact with temperature); photochromic materials (which interact with light intensity or brightness); hydrochromic materials (which interact with water); electrochromic materials (which interact with electrical current); materials with shape “memory” (which change their shape with different stimuli); between others. Thus, new visual effects can be obtained in the visible and invisible spectrum observed by the thermal imaging camera, enabling/allowing different interactions, illusions, movements, textile manipulations that can enhance the impact of the varying textile patterns in different contexts (artistic and military).

Otherwise, the conventional material (knitted fabric Jersey 100% Polyester; white color; 153.39 g/m^2^; 0.42 mm; 26.9 Tex; 15 wales/cm; 20 courses/cm; 0.42 mm; 0.76 ε) and the metallic pigment tested (formula: 80 g Hydra Clear for Metallic (clear base of the brand Virus^®^, Water Based Inks, Bergamo, Italy) with 20 g Copper Powder (metallic powder of the brand Virus^®^, Water Based Inks, Bergamo, Italy), such as the printing and sewing technique applied in this work, proved to be a practical and economical solution to be reproduced in different textile products, however, it was also revealed as a starting point in the exploration of the textile surface with thermal camouflage functionality.

To sum up, there are no limits to the creation of new perspectives in clothing, combining the different visible and invisible spectra. The work and concept developed can be applied and studied in different aspects, such as in the exploration of different environments and camouflage backgrounds, in the exploration of different atmospheric conditions such as extreme adverse conditions, in the exploration of different types of bodies and silhouettes; in the exploration and creation of new prototypes and experimentation of new textile materials and textile manipulation techniques, in the exploration and interconnection with different arts and types of exhibition, in the exploration and interconnection with visual camouflage, envisaging a simultaneous thermal and visual camouflage functionality, with a view to developing the best possible camouflage.

## Data Availability

No new data were created or analyzed in this study.
